# Supplementary material for: The complete sequence and comparative analysis of ape sex chromosomes
Source: Nature. 2024 May 29;630(8016):401–11. doi: 10.1038/s41586-024-07473-2 (PMC11168930; doi:10.1038/s41586-024-07473-2)
Supplement: Supplementary file 2 — Reporting Summary [file 41586_2024_7473_MOESM2_ESM.pdf]

nature portfolio

Corresponding author(s): Kateryna D. Makova, Evan E. Eichler, & Adam M. Phillippy

Last updated by author(s): Apr 1, 2024

Reporting Summary

Nature Portfolio wishes to improve the reproducibility of the work that we publish. This form provides structure for consistency and transparency in reporting. For further information on Nature Portfolio policies, see our Editorial Policies and the Editorial Policy Checklist.

Statistics

For all statistical analyses, confirm that the following items are present in the figure legend, table legend, main text, or Methods section.

n/a	Confirmed	
☐	☒	The exact sample size (*n*) for each experimental group/condition, given as a discrete number and unit of measurement
☐	☒	A statement on whether measurements were taken from distinct samples or whether the same sample was measured repeatedly
☐	☒	The statistical test(s) used AND whether they are one- or two-sided<br>*Only common tests should be described solely by name; describe more complex techniques in the Methods section.*
☒	☐	A description of all covariates tested
☐	☒	A description of any assumptions or corrections, such as tests of normality and adjustment for multiple comparisons
☐	☒	A full description of the statistical parameters including central tendency (e.g. means) or other basic estimates (e.g. regression coefficient) AND variation (e.g. standard deviation) or associated estimates of uncertainty (e.g. confidence intervals)
☐	☒	For null hypothesis testing, the test statistic (e.g. *F*, *t*, *r*) with confidence intervals, effect sizes, degrees of freedom and *P* value noted<br>*Give P values as exact values whenever suitable.*
☒	☐	For Bayesian analysis, information on the choice of priors and Markov chain Monte Carlo settings
☒	☐	For hierarchical and complex designs, identification of the appropriate level for tests and full reporting of outcomes
☐	☒	Estimates of effect sizes (e.g. Cohen's *d*, Pearson's *r*), indicating how they were calculated

Our web collection on statistics for biologists contains articles on many of the points above.

Software and code

Policy information about availability of computer code

Data collection

Raw data collection relied on software installed on various instruments (e.g., a sequencing machine), which would be available to any user of such an instrument, and, when relevant, we have noted version numbers of such software packages in the text alongside the information on instrumentation and other methods. No new data collection software was created for this work, though many mundane data organization and sharing tasks (e.g., renaming and group files or sharing data with collaborators over the internet) were performed that were not documented because they are relatively commonplace and uninteresting. All subsequent processing of raw data is considered part of the "analysis" of the data, and all analyses are extensively documented in the text (especially in the methods and supplementary information). Please see our response in the "Data analysis" portion of the "Software and code" availability of this form for more detail.

Data analysis

Analyses primarily relied on previously-published, open-source software, and such software packages are mentioned in the text (especially the methods and supplementary information), with a version number and citation. No new algorithms or software packages were created for this study, though some custom scripts were created and run for certain tasks. When the methods for a particular analysis would be difficult to replicate from the text with listed options and/or commands alone, more detailed code is provided in a publicly-available repository on GitHub (https://github.com/makovalab-psu/T2T_primate_XY) and at Zenodo (https://doi.org/10.5281/zenodo.10680008). As necessary, external scripts and programs are also linked through this GitHub repository.

All tools and versions from paper listed below:

Counting rDNA Copy Number analysis on GitHub:

jellyfish 2.2.10
samtools 1.3.1

htslib 1.3.1
bedtools v2.29.0
Denovo genes
Github
Python=3.8.10

Python packages:
Bio=1.81,
pandas=1.5.3

methylation analysis on GitHub:
R version 4.3.2
R packages:
cowplot=1.1.3
GenomicRanges=1.54.1
ggplot2=3.5.0,
ggsignif=0.6.4,
scales=1.3.0

Multicopy genes analysis on GitHub:
Python 3.12.2

Python packages:
bcbio-gff==0.7.0
biopython==1.81
gspread==5.9.0
pandas==2.0.1
seaborn==0.12.2
matplotlib==3.7.1

palindromes analysis on GitHub:
Conda environment:

```
name: alignment
channels:
  - anaconda
  - bioconda
  - defaults
dependencies:
  - _libgcc_mutex=0.1
  - _openmp_mutex=5.1
  - bamtools=2.5.1
  - bedtools=2.30.0
  - bioawk=1.0
  - blas=1.0
  - blast=2.5.0
  - blat=35
  - boost=1.73.0
  - bzip2=1.0.8
  - c-ares=1.19.0
  - ca-certificates=2023.01.10
  - curl=7.88.1
  - emboss=6.6.0
  - expat=2.4.9
  - fasta-splitter=0.2.6
  - fontconfig=2.14.1
  - freetype=2.12.1
  - giflib=5.2.1
  - gsl=2.6
  - icu=58.2
  - jemalloc=5.2.1
  - jpeg=9e
  - k8=0.2.5
  - krb5=1.19.4
  - ld_impl_linux-64=2.38
  - lerc=3.0
  - libboost=1.73.0
  - libcurl=7.88.1
  - libdeflate=1.17
  - libedit=3.1.20221030
  - libev=4.33
  - libffi=3.4.4
  - libgcc-ng=11.2.0
  - libgd=2.3.3
  - libgfortran-ng=11.2.0
  - libgfortran5=11.2.0
  - libgomp=11.2.0
```

- libnghttp2=1.46.0
- libnsl=2.0.0
- libopenblas=0.3.21
- libpng=1.6.39
- libssh2=1.10.0
- libstdcxx-ng=11.2.0
- libtiff=4.5.0
- libuuid=1.41.5
- libwebp=1.2.4
- libwebp-base=1.2.4
- libxml2=2.10.3
- lz4-c=1.9.4
- mashmap=2.0
- meryl=1.3
- minimap2=2.17
- ncurses=6.4
- nomkl=3.0
- numpy=1.23.5
- numpy-base=1.23.5
- openblas=0.3.21
- openblas-devel=0.3.21
- openssl=1.1.1u
- perl=5.32.1
- perl-constant=1.33
- perl-exporter=5.72
- perl-file-util=4.201720
- perl-lib=0.63
- pip=23.0.1
- py-boost=1.73.0
- python=3.10.11
- readline=8.2
- samtools=1.6
- seqtk=1.3
- setuptools=67.8.0
- sqlite=3.41.2
- tk=8.6.12
- tzdata=2023c
- wfmash=0.7.0
- wheel=0.38.4
- winnowmap=2.03
- xz=5.2.10
- zlib=1.2.13
- zstd=1.5.5

palindrover_maf_align analysis on GitHub:
Python 3.11.0+
Lastz 1.04.22

Python packages
palindrover 0.1.5 (included)
maf_alignments 0.1.0

Phylogenetic inference analysis on GitHub:
Python=3.9.11

Python packages:
Bio=1.79
pandas=1.4.1
numpy=1.21.2

Sequence classes analysis on Github:
Bedtools=v2.30.0
Samtools=1.6
Python=3.10.11
Blast=2.5.0+
Circos=v 0.69-8 | 15 Jun 2019 | Perl 5.032001
xtr_search
Github
R=4.3.0
R packages
ggplot=3.4.3
cowplot=1.1.1

Sequencing analysis in Supplementary methods:
Guppy=6.0.0+

Assembly
Suppl.
methods
Verrko v1.1
Bandage v0.8.1
BangageNG v2022.09
IGV v2.25.4
Merqury v1.3
Hifiasm v0.16.1-r375
MashMap v2.0
Flye=v2.9-b1768
BWA-MEM2 v2.2.1
Winnowmap2 v2.03
Meryl v1.03
Non-B annot
Suppl.
methods
non-B_gfa (commit 2f5a24f)
Alignments
Suppl.
methods
Minimap2 2.24
Lastz 1.04.22

Phylogenetic analysis in Supplementary methods:
Cactus 2.6.0
Hal2maf 2.2
IQTree 2.0.3

Substitution Frequency analysis in Supplementary methods:
Cactus 2.2.0
Maftools Oct 20 2022
phast 1.5

Gene annotations (NCBI) analysis in Supplementary methods:
BUSCO 4.0.2

Gene annotation (UCSC) analysis in Supplementary methods:
Cactus 2.6.0
Minimap2
CAT 2.2.1
Liftoff 1.6.3

Repeat and satellite annotation analysis in Supplementary methods:
RepeatMaske v4.1.2-p1
Dfam 3.6 [data]
Repbase v20181026
Bedtools 2.29.0
TRF v4.09
ULTRA v1.0
Minidot v2016
Rideogram v0.2.2

Sequence Classes analysis in Supplementary methods:
SEDEF v1.1
TRF v4.0.9
RepeateMasker v4.1.2-p1
Windowmaser v2.2.22
Blast 2.5.0+
palindrover 20230615

Segmental duplications analysis in Supplementary methods:
SEDEF v1.1
TRF v4.0.9
Windowmasker v2.2.22
Minimap2 2.24
Blast 2.5.0+
SV's
Suppl.
methods
Minimap2 2.24
Bedtools 2.29.2
Rustybam v0.1.29

rDNA arrays analysis in Supplementary methods:
Ribotin=v1.1.0

T2T-Polish=v1.0

Methylation analysis in Supplementary methods:
Meryl v1.3
Guppy v6.3.8
Pbccs v6.4.0
Primrose v1.3.0
Winnowmapt v2.03
Samtools 1.17
ModBam2bed=v0.6.2
featureCounts v2.0.6

Diversity analysis in Supplementary methods:
BWA-MEM v0.7.17
GATK v4.4.0.0
VCFtools v0.1.16

Y phylogeny TMRCA analysis in Supplementary methods:
VCFtoold v0.1.16
BEAST v1.10.4
IQ-TREE 1.6.12
Tree-Annotator 1.10.4
FigTree v1.4.4

Chimp subspecies identification analysis in Supplementary notes S2:
Seqtk v1.4
Mash (commit 41ddc61)
Clustal Omega v1.2.4
Bowtie2 v2.5.1
Samtools v1.17
Mashmap v2.0
tRNAscan-SE v2.0.11
nucmer v4.0.0rc1

Bonobo PAR2 and Ariel satellites analysis in Supplementary notes S3:
BLAST+ v2.14.0
Winnowmap v2.03
SAMtools v1.18
Mashmap v2.0

XTR analysis in Supplementary notes S4:
R v4.3.0
ggplot2 v3.4.3
cowplot v1.1.1

De novo genes analysis in Supplementary notes S11:
BLAST 2.12.0 (accessed online Oct 2023)
NetSurfP-3.0
flDPnn (has no version number)
AGGRESCAN (has no version number)
Protein-Sol (has no version number)
ESMFold (has no version number)
All tools used/accessed in October 2023)

Selection analysis of ancestral (X-degenerate) Y genes using diversity data in Supplementary notes S13:
Bcftools 1.9
CACTUS 3.1.20211107152837
vcfR 1.14.0
Ape 5.7
R 4.3.0
Hal2maf 2.2
IQTree 2.0.3
AGAT 1.2.0
MACSE 2.07
HyPhy 2.5.50
Gblocks 0.91b
aBSREL 2.23.0

For manuscripts utilizing custom algorithms or software that are central to the research but not yet described in published literature, software must be made available to editors and reviewers. We strongly encourage code deposition in a community repository (e.g. GitHub). See the Nature Portfolio guidelines for submitting code & software for further information.

Data

Policy information about availability of data

All manuscripts must include a data availability statement. This statement should provide the following information, where applicable:

- Accession codes, unique identifiers, or web links for publicly available datasets
- A description of any restrictions on data availability
- For clinical datasets or third party data, please ensure that the statement adheres to our policy

The raw sequencing data generated in this study have been deposited in the Sequence Read Archive under BioProjects PRJNA602326, PRJNA902025, PRJNA976699-PRJNA976702, and PRJNA986878-PRJNA986879. The genome assemblies and NCBI annotations are available from GenBank/RefSeq (see Table S46 for accession numbers). The CAT/Liftoff annotations are available in a UCSC Genome Browser Hub: https://cgl.gi.ucsc.edu/data/T2T-primates-chrXY/. The reference genomes, alignments and variant calls are also available within the NHGRI AnVIL: https://anvil.terra.bio/#workspaces/anvil-dash-research/AnVIL_Ape_T2T_chrXY. The alignments generated for this project are available at: https://www.bx.psu.edu/makova_lab/data/APE_XY_T2T/ and https://public.gi.ucsc.edu/~hickey/hubs/hub-8-t2t-apes-2023v1/8-t2t-apes-2023v1.hal (with the following additional information: https://public.gi.ucsc.edu/~hickey/hubs/hub-8-t2t-apes-2023v1/8-t2t-apes-2023v1.README.md) Additional Data Files include human-specific structural variant coordinates (File 1), sequence class coordinates (File 2), palindrome coordinates (File 3), RNA-Seq and IsoSeq datasets used for gene annotations (File 4), and manual annotations of Y ampliconic genes (File 5). Primary data related to the cytogenetic evaluation of the rDNA is deposited in the Stowers Institute Original Data Repository under accession LIBPB-2447: https://www.stowers.org/research/publications/libpb-2447. C-values used for genome size estimates (see Supplemental Methods) were taken from the Animal Genome Size Database (https://www.genomesize.com) as found on Genome on a Tree (https://goat.genomehubs.org). Existing reference assemblies used for comparison can be found under the following accessions on NCBI: GCA_013052645.3 (bonobo, Mhudiblu), GCA_015021855.1 (bonobo; chrY), GCF_002880755.1 (chimpanzee, Clint), GCF_008122165.1 (gorilla, Kamilah), GCA_015021865.1 (gorilla, Jim; chrY), GCA_009914755.4 (human, T2T-CHM13v2.0), GCF_002880775.1 (Sumatran orangutan, Suzie), and GCA_015021835.1 (Sumatran orangutan; chrY). Short read datasets from other ape individuals used for mapping and diversity analyses were obtained from NCBI under the following accessions: SRP018689, ERP001725, ERP016782, and ERP014340 (see Table S42).

Research involving human participants, their data, or biological material

Policy information about studies with human participants or human data. See also policy information about sex, gender (identity/presentation), and sexual orientation and race, ethnicity and racism.

Reporting on sex and gender	N/A: No human participants were part of this study
Reporting on race, ethnicity, or other socially relevant groupings	*Please specify the socially constructed or socially relevant categorization variable(s) used in your manuscript and explain why they were used. Please note that such variables should not be used as proxies for other socially constructed/relevant variables (for example, race or ethnicity should not be used as a proxy for socioeconomic status).*<br>*Provide clear definitions of the relevant terms used, how they were provided (by the participants/respondents, the researchers, or third parties), and the method(s) used to classify people into the different categories (e.g. self-report, census or administrative data, social media data, etc.)*<br>*Please provide details about how you controlled for confounding variables in your analyses.*
Population characteristics	*Describe the covariate-relevant population characteristics of the human research participants (e.g. age, genotypic information, past and current diagnosis and treatment categories). If you filled out the behavioural & social sciences study design questions and have nothing to add here, write "See above."*
Recruitment	*Describe how participants were recruited. Outline any potential self-selection bias or other biases that may be present and how these are likely to impact results.*
Ethics oversight	*Identify the organization(s) that approved the study protocol.*

Note that full information on the approval of the study protocol must also be provided in the manuscript.

Field-specific reporting

Please select the one below that is the best fit for your research. If you are not sure, read the appropriate sections before making your selection.

☒ Life sciences ☐ Behavioural & social sciences ☐ Ecological, evolutionary & environmental sciences

For a reference copy of the document with all sections, see nature.com/documents/nr-reporting-summary-flat.pdf

Life sciences study design

All studies must disclose on these points even when the disclosure is negative.

Sample size	We analyzed one cell line per species as an initial investigation of sex chromosomes in great apes. No sample size calculation was done.
Data exclusions	No data were excluded from the analyses
Replication	We did not replicate our experiments except for (1) droplet digital PCR that was conducted in triplicates, and (2) FISH experiments for rDNA probing (conducted in 20 replicates). All attempts at replication were successful. Most additional analyses were computational and thus were

	not conducted in replicates.
Randomization	N/A because we only used one sample per species
Blinding	N/A because we only used one sample per species

Reporting for specific materials, systems and methods

We require information from authors about some types of materials, experimental systems and methods used in many studies. Here, indicate whether each material, system or method listed is relevant to your study. If you are not sure if a list item applies to your research, read the appropriate section before selecting a response.

Materials & experimental systems

n/a	Involved in the study
☐	☒ Antibodies
☐	☒ Eukaryotic cell lines
☒	☐ Palaeontology and archaeology
☒	☐ Animals and other organisms
☒	☐ Clinical data
☒	☐ Dual use research of concern
☒	☐ Plants

Methods

n/a	Involved in the study
☒	☐ ChIP-seq
☒	☐ Flow cytometry
☒	☐ MRI-based neuroimaging

Antibodies

Antibodies used	1. rabbit polyclonal anti-UBF, Novus Biologicals, cat.# NBP1-82545<br>2. goat anti-rabbit Alexa Fluor 647, Thermo Fisher Scientific
Validation	1. Novus Biologicals website lists seven publications using this antibody, including one publication that uses it for immunofluorescence.<br>2. Thermo Fisher's website lists many publications using this antibody. The website also specifies that "the sensitivity and specificity of each lot is confirmed using ELISA" and there is "minimal cross-reactivity with mouse, rat, human, bovine, guinea pig, and donkey IgG".

Eukaryotic cell lines

Policy information about cell lines and Sex and Gender in Research

Cell line source(s)	KB8711 (San Diego Zoological Society), AG18354 (Coriell), AG06213 (Coriell), AG05252 (San Diego Zoological Society), KB3781 (San Diego Zoological Society), Jambi (Oregon Health and Science University)
Authentication	Each cell line was authenticated with karyotyping. We authenticated species for orangutans as described in Note S1 and and subspecies for chimpanzee as described in Note S2
Mycoplasma contamination	The cell lines were not tested for mycoplasma contamination in our laboratories, but they were most likely tested for such contamination by cell line providers
Commonly misidentified lines<br>(See ICLAC register)	No commonly misidentified cell lines were used.

Plants

Seed stocks	N/A: Plants were not part of this study
Novel plant genotypes	*Describe the methods by which all novel plant genotypes were produced. This includes those generated by transgenic approaches, gene editing, chemical/radiation-based mutagenesis and hybridization. For transgenic lines, describe the transformation method, the number of independent lines analyzed and the generation upon which experiments were performed. For gene-edited lines, describe the editor used, the endogenous sequence targeted for editing, the targeting guide RNA sequence (if applicable) and how the editor was applied.*
Authentication	*Describe any authentication procedures for each seed stock used or novel genotype generated. Describe any experiments used to assess the effect of a mutation and, where applicable, how potential secondary effects (e.g. second site T-DNA insertions, mosiacism, off-target gene editing) were examined.*
